# Taxonomy of the *Leptogenys modiglianii* species group from southeast Asia (Hymenoptera, Formicidae, Ponerinae)

**DOI:** 10.3897/zookeys.651.10336

**Published:** 2017-02-02

**Authors:** Kôichi Arimoto

**Affiliations:** 1 Entomological Laboratory, Graduate School of Bioresource and Bioenvironmental Sciences, Kyushu University, Hakozaki 6-10-1, Fukuoka, 812–8581 Japan

**Keywords:** Ants, new species, Oriental region, Ponerini, redescription, taxonomic revision

## Abstract

*Leptogenys breviloba* **sp. n.**, *Leptogenys curva* **sp. n.**, *Leptogenys itoi* **sp. n.**, *Leptogenys kanaoi* **sp. n.**, *Leptogenys malayana* **sp. n.**, and *Leptogenys modiglianii* Emery, 1900 are described from southeast Asia. The *Leptogenys modiglianii* species group is proposed on the basis of similarities among the six species. An identification key to species in this group from southeast Asia is provided.

## Introduction

The genus *Leptogenys* Roger, 1861 is one of the largest ant genera, and contains 302 species and 25 subspecies throughout the tropical and subtropical regions (Bolton 2016). In the Oriental region, approximately 70 *Leptogenys* species have been described. They have not been comprehensively studied, although Xu and He (2015) provided a preliminary key to the known Oriental species. There are no species groups well defined on the basis of worker morphology in this region. *Leptogenys modiglianii* Emery, 1900 was described from Engano Island, which is located southwest of Sumatra, Indonesia. Subsequently, Mann (1919) provided a key to species related to *Leptogenys modiglianii* in the Australian region, but no additional species in this group have been found in the Oriental region. *Leptogenys modiglianii* was not treated in Xu and He (2015). In this paper, the *Leptogenys modiglianii* species group is proposed, and all species treated here are described from southeast Asia. An identification key is provided for all the species.

## Materials and methods

Depositories of the type specimens are as follows:

BLKU
Laboratory of Biology, Faculty of Agriculture, Kagawa University, Takamatsu, Japan.


BMNH
The Natural History Museum, London, United Kingdom.


ELKU
Entomological Laboratory, Kyushu University, Fukuoka, Japan.


FDS
Forest Department Sarawak, Kuching, Malaysia.


SKYC
Seiki Yamane Collection, Kagoshima, Japan.


MSNG
Museo Civico di Storia Naturale Giacomo Doria, Genova, Italy.


NHMW
Naturhistorisches Museum Wein, Vienna, Austria.


Colony codes and depositories are shown in round and square brackets, respectively.

Photographs of specimens were taken with a Canon EOS 7D digital camera with a Canon macro photo lens MP-E 65mm (Canon Inc., Tokyo, Japan) and then combined using the CombineZM software. General structures of specimens were observed with an Olympus-SZX9 stereomicroscope (Olympus Corporation, Tokyo, Japan). Maps were generated using DIVA-GIS 7.5.0. Digital images of photographs and maps were edited with Adobe Photoshop 7.0 (Adobe, San Jose, CA, USA).

### Measurements and indices

**Figure 1.**
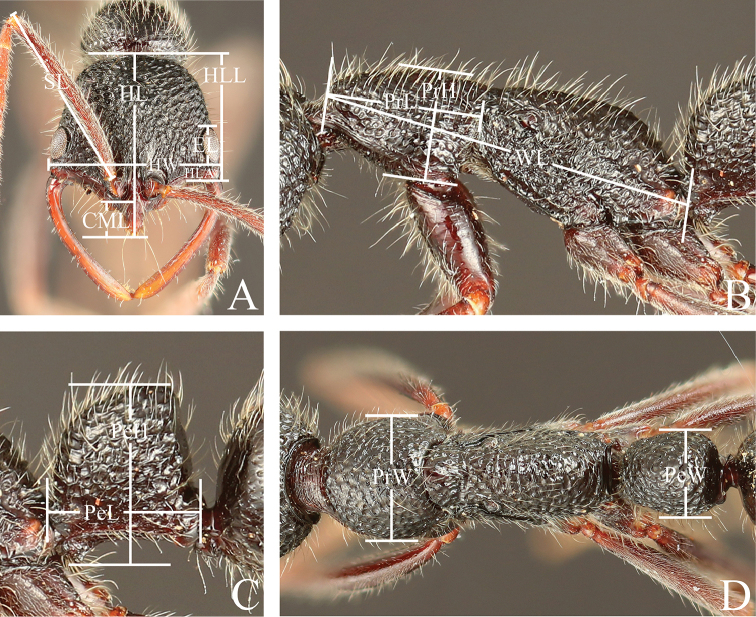
Measurements of worker of the *Leptogenys modiglianii* species group. **A** Head, full-face view **B** lateral view **C** petiole, lateral view **D** dorsal view. Abbreviations: HL Head length, HLL Head lateral margin length, HLA Anterior head length, HW Head width, CML Clypeal median lobe length, SL Scape length, EL Eye length, PrL Pronotum length, PrH Pronotum height, PrW Pronotum width, WL Weber’s length, PeL Petiole length, PeH Petiole height, PeW Petiole width.

**Figure 2.**
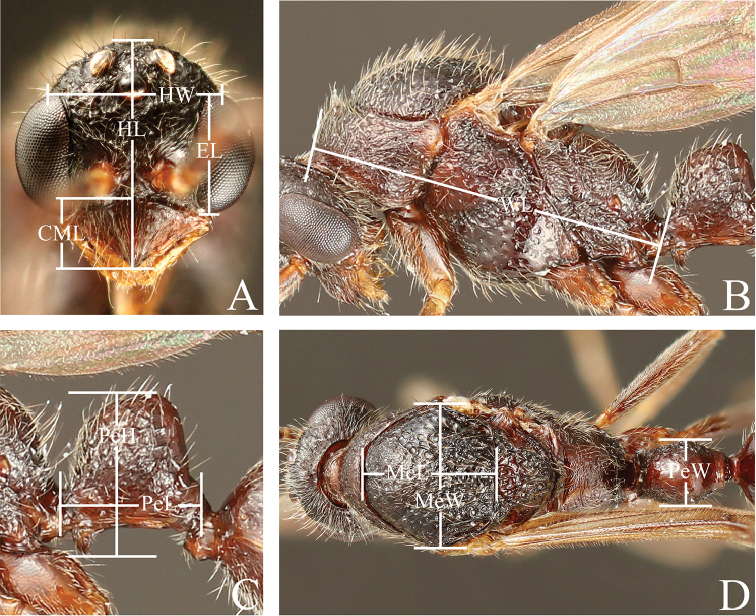
Measurements of male of the *Leptogenys modiglianii* species group. **A** Head, full-face view **B** lateral view **C** petiole, lateral view **D** dorsal view. HL Head length, HW Head width, CML Clypeal median lobe length, EL Eye length, MeL Mesoscutum length, MeW Mesoscutum width, WL Weber’s length, PeL Petiole length, PeH Petiole height, PeW Petiole width.

Measurements are shown in millimeters and were made with a micro ruler (MR-2, minimum scale value: 0.05 mm). The following measurements are visualized in Figures 1 and 2. The ratios were calculated from measurements and are denominated indices.

HL
Head length: in full-face view, the maximum length of the head excluding the mandible, measured from the anterior margin of the clypeus to the nuchal carina.

HLL
Head lateral margin length: in full-face view, the head length measured from the mandible base to the nuchal carina.

HLA
Anterior head length: in full-face view, the head length measured from the mandible base to the anterior edge of the eye.

HW
 Head width: in full-face view, the maximum width of the head excluding the eyes.

CML
 Clypeal median lobe length: in full-face view, the straight-line length measured from the anterior margin of the clypeus to the anterior margin of the torulus.

CI
 Cephalic index: HW/HL × 100.

CLI
 Clypeus index: CML/HL × 100.

SL
 Scape length: the straight-line length of the first antennal segment, excluding the neck and the basal condyle.

SI
 Scape index: SL/HW × 100.

EL
 Eye length: in full-face view, the vertical line length of the compound eye.

OI
 Ocular index: EL/HLL × 100.

PrL
 Pronotum length: in profile, the diagonal length of the pronotum, measured from the anterior margin of the pronotum excluding the collar to the posterior extremity of the pronotum.

PrH
 Pronotum height: in profile, the maximum height of the pronotum, measured from the posterior base of the lateral margin of the pronotum to the highest point of the pronotum.

PrW
 Pronotum width: in dorsal view, the maximum width of the pronotum.

MeL
 Mesoscutum length: in dorsal view, the maximum length of the mesoscutum.

MeW
 Mesoscutum width: in dorsal view, the maximum width of the mesoscutum.

WL
 Weber’s length: in profile, the diagonal length of the mesosoma, measured from the anterior margin of the pronotum excluding the collar to the posterior extremity of the propodeal lobe.

PeL
 Petiole length: in profile, the maximum length of the petiole, measured from the anteriormost margin to the posteriormost margin of the petiole, including the peduncle.

PeH
 Petiole height: in profile, the height of the node, measured from the apex of the subpetiolar process to the highest point of the node.

PeW
 Petiole width: in dorsal view, the maximum width of the node.

LPI
 Lateral petiole index: PeH/PeL × 100.

DPI
 Dorsal petiole index: PeW/PeL × 100.

## Taxonomy

### The *modiglianii* species group (proposed here)

**Species included in southeast Asia.** Six species: *Leptogenys breviloba* sp. n., *Leptogenys curva* sp. n., *Leptogenys itoi* sp. n., *Leptogenys kanaoi* sp. n., *Leptogenys malayana* sp. n., *Leptogenys modiglianii* Emery, 1900

**Figure 3.**
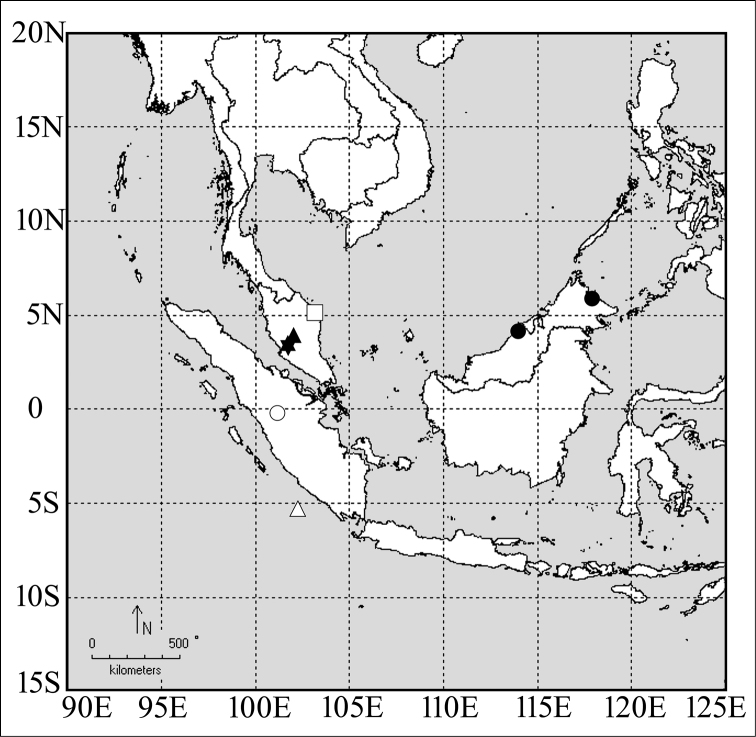
Collecting localities of species of the *modiglianii* species group. Black star: *Leptogenys breviloba* and *Leptogenys itoi*, white circle: *Leptogenys curva*, black triangle: *Leptogenys itoi*, black circle: *Leptogenys kanaoi*, white rectangle: *Leptogenys malayana*, white triangle: *Leptogenys modiglianii*.

**Distribution** (Fig. 3). Malaysia (Peninsular Malaysia, Borneo), Indonesia (Sumatra, Engano Island).

**Figure 4.**
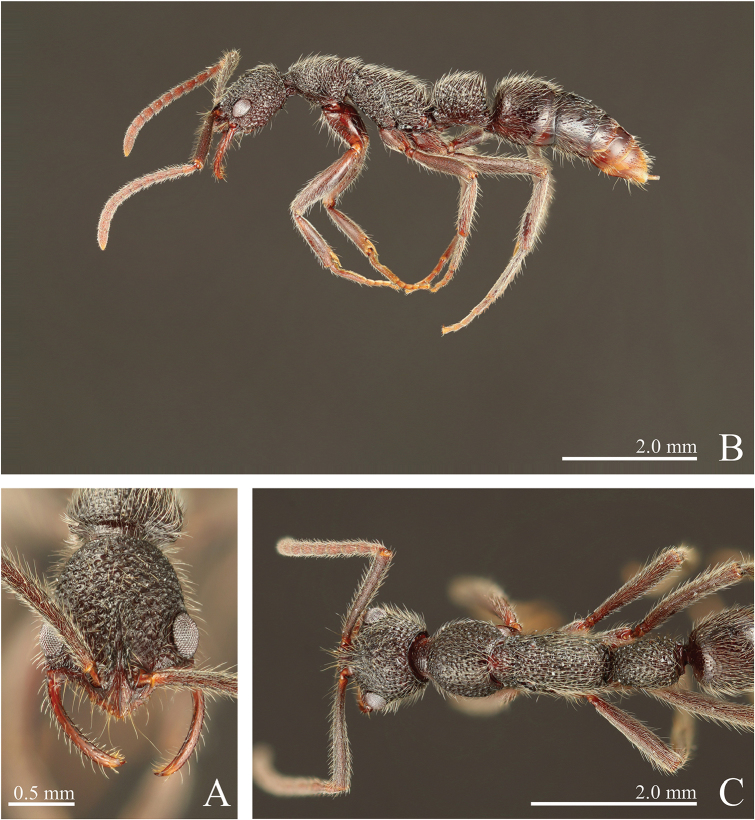
*Leptogenys breviloba* sp. n., holotype, worker. **A** head, full-face view **B** lateral view **C** dorsal view.

***Worker*** (Figs 4, 6, 8, 11, 13–19). **Diagnosis.** Head widest just posterior to clypeus excluding eyes. Mandible sickle-shaped, elongate, without denticles. Anterior edge of torulus located anterior to mandible bases. Clypeus short, with apical median extension. Eye situated just posterior to clypeus, breaking outline of lateral head margin. Hypostomal teeth large, visible in full-face view. In profile, petioler node rectangular to fan-shape; subpetiolar process consisting of high anterior lobe and thin posterior extension. Body surface mostly areolate-rugose, extensively with standing long hairs.

**Description.** In full-face view, head either longer than wide or almost as long as wide (CI: 77-101), broadening anteriad, widest just posterior to clypeus excluding eyes; posterior margin broadly convex. Mandible sickle-shaped, elongate, curved near base and becoming straight apically or curved throughout, without denticles, subapical tooth either absent or present near apical tooth; internal margin with narrow lamella; large gap present between mandibular shaft and clypeus; mandibles with only their apical portions crossing each other when fully closed. Eye large (OI: 26–39), situated just posterior to clypeus, breaking outline of lateral margin of head. Antennal scape almost as long as width of head or longer (SI: 99-121), surpassing posterior margin of head by one-sixth to two-fifths of its length; antennomere III longer than II; antennomere XII shorter than III, longer than XI. Anterior edge of torulus located anterior to mandible base. Clypeus short, with longitudinal median carina; anterolateral margin broadly concave, either with or without lateral lobe; apical median extension (CLI: 12–21) with convex or truncate apex, fringed by narrow translucent lamella, with pair of peg-like setae. Hypostomal teeth large, visible in full-face view. Pronotum in profile longer than high, in dorsal view shorter than wide or longer. Mesonotum in dorsal view shorter than wide. Metanotal groove distinctly visible, weakly to strongly impressed. Meso-metapleural sulcus present. In profile, propodeal dorsum convex to straight, posteriorly rounding into declivity, longer than length of propodeal declivity; metapleural-propodeal sulcus shallow. Petiole in profile longer than high or shorter (LPI: 94–118), in dorsal view longer than wide (DPI: 71–87); node in profile either rectangular (dorsal face subhorizontal) or fan-shape (dorsal face distinctly sloping anteriad), highest posteriorly, with anterior and dorsal faces forming continuous curve or anterior face of node ventrally vertical and dorsally forming continuous curve with dorsum, in dorsal view widening posteriad, with lateral margin broadly convex; posterior face flat, abruptly separated from lateral face, slightly inclined anteriad. Subpetiolar process consisting of high anterior lobe and thin posterior extension; anterior lobe fan-shaped, with continuously curved anteroventral margin and concave posterior margin. Anterior face of gastral tergite I flat; in profile, anteroventral edge of gastral sternite I shallowly indented just posterior to prora; in profile, prora long-rectangular, low; constriction between gastral segments I and II distinct.

Head, mesosoma, and petiole mostly areolate-rugose, superimposed with small piligerous punctures. Antennal scape and legs densely punctuated. Clypeus with oblique to longitudinal striae. Propodeal declivity with transverse crests. Postgenal bridge and prosternum sparsely with circular depressions, medially smooth and without punctures. Mesosternum smooth and without punctures, sometime with transverse striate anteriorly and posteriorly. Ventrolateral and posterior faces of petiolar node smooth; subpetiolar process finely striate. Anterior face of gastral tergite I smooth; gastral segment I with scalloped depressions that are deepest anteriorly and sloping posteriad; segment II smooth or covered with scalloped depressions; constriction between gastral segments I and II finely scrobiculate; segments III–V smooth.

Body black and tinged with red, shiny. Hairs of various lengths, scattered, white-yellow; long hairs standing; short pubescence decumbent; antenna and legs with dense short pubescence, mixed with scattered long hairs.

**Figure 5.**
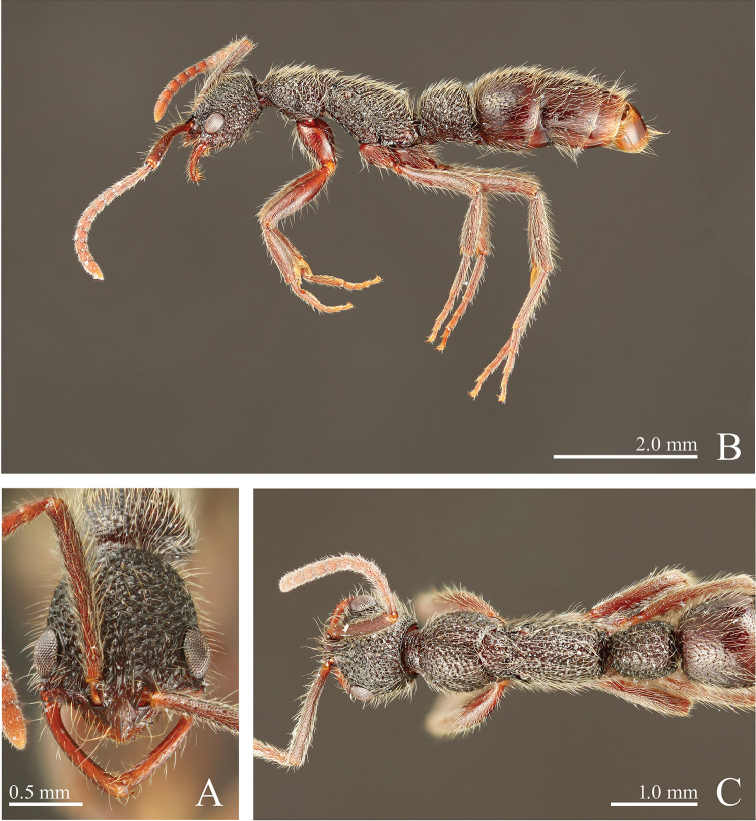
*Leptogenys breviloba* sp. n., paratype, queen; gastral apical segments removed. **A** head, full-face view **B** lateral view **C** dorsal view.

***Queen*** (Figs 5, 7, 9, 12). In general structure the queen is very similar to the worker (ergatoid queen) and lacks ocelli; it differs from the worker in having a higher and wider petiole (LPI: 105–132; DPI: 79–94) and voluminous gaster.

***Male*** (Fig. 10). Only known from *Leptogenys itoi* (see below).

**Figure 6.**
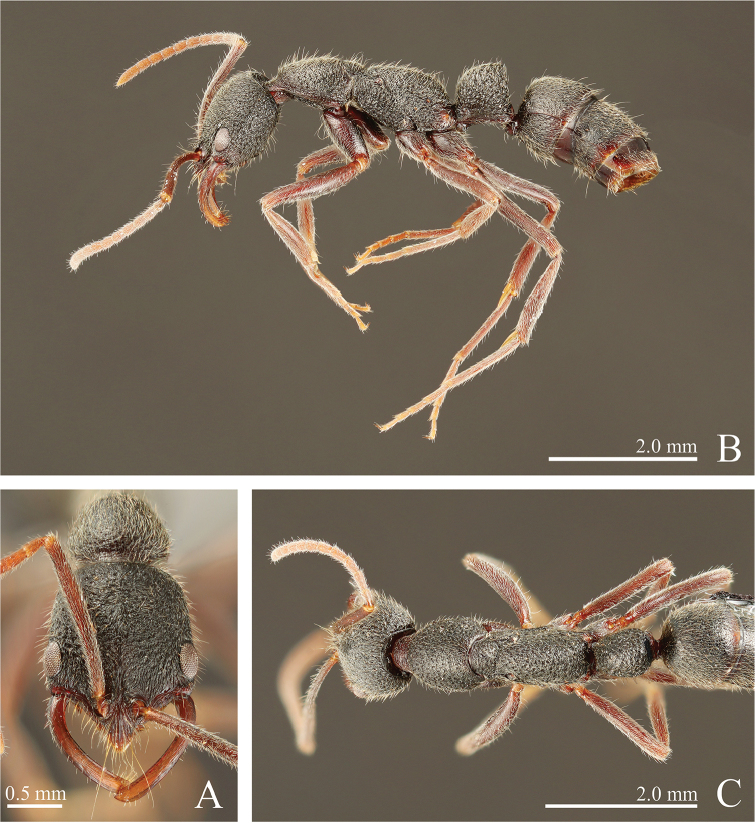
*Leptogenys curva* sp. n., holotype, worker; gastral apical two segments removed. **A** head, full-face view **B** lateral view **C** dorsal view.

**Figure 7.**
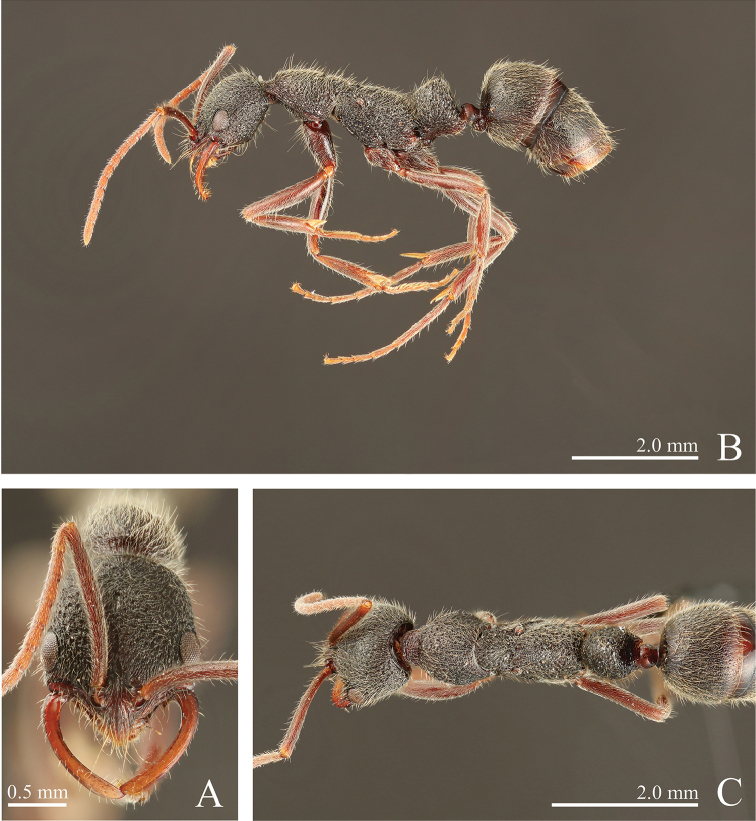
*Leptogenys curva* sp. n., paratype, queen; gastral apical two segments removed. **A** head, full-face view **B** lateral view **C** dorsal view.

**Figure 8.**
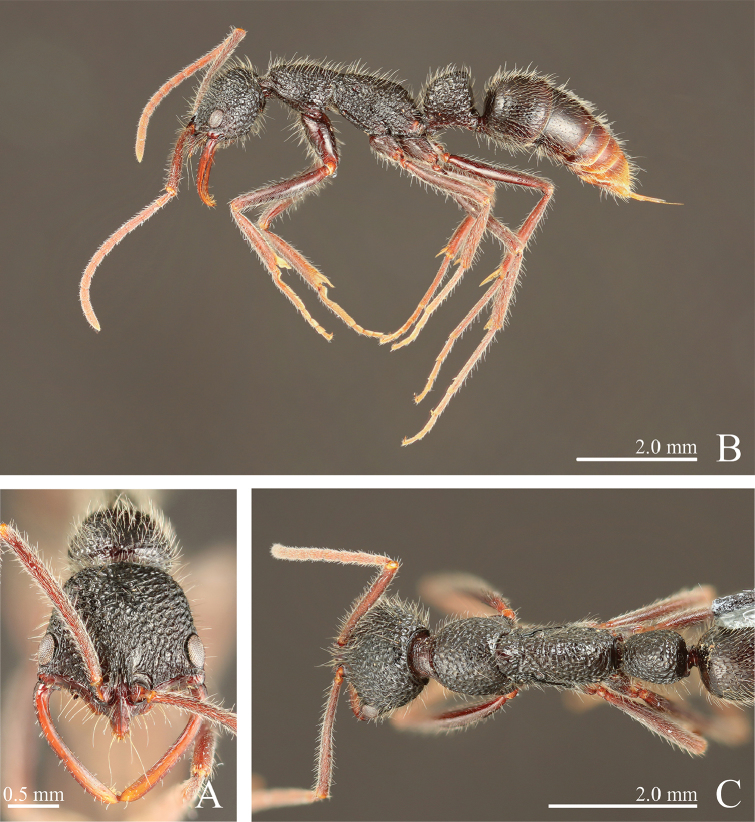
*Leptogenys itoi* sp. n., holotype, worker. **A** head, full-face view **B** lateral view, **C** dorsal view.

**Figure 9.**
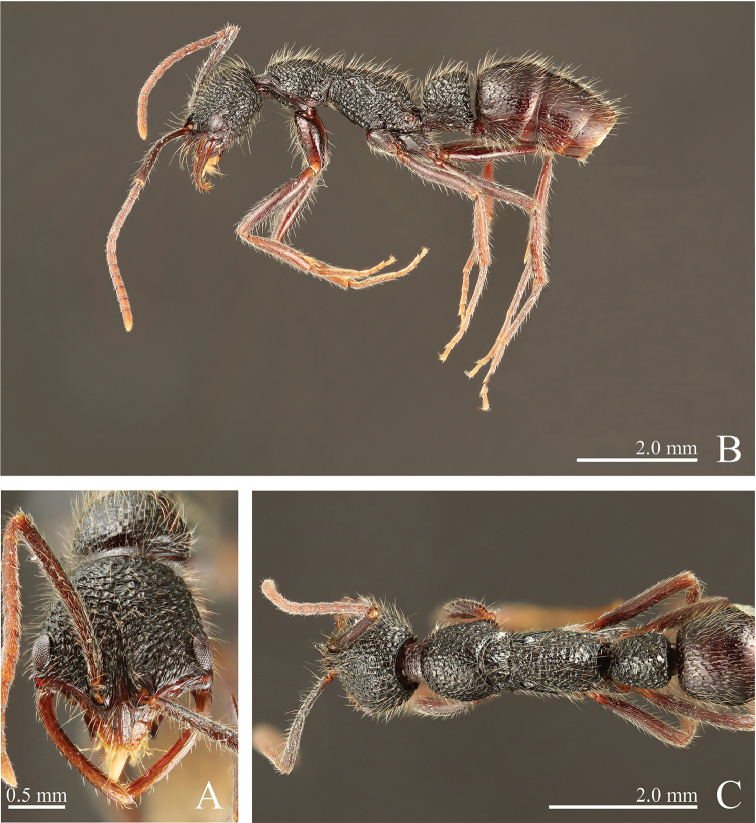
*Leptogenys itoi* sp. n., paratype, queen; gastral apical two segments removed. **A** head, full-face view **B** lateral view **C** dorsal view.

**Figure 10.**
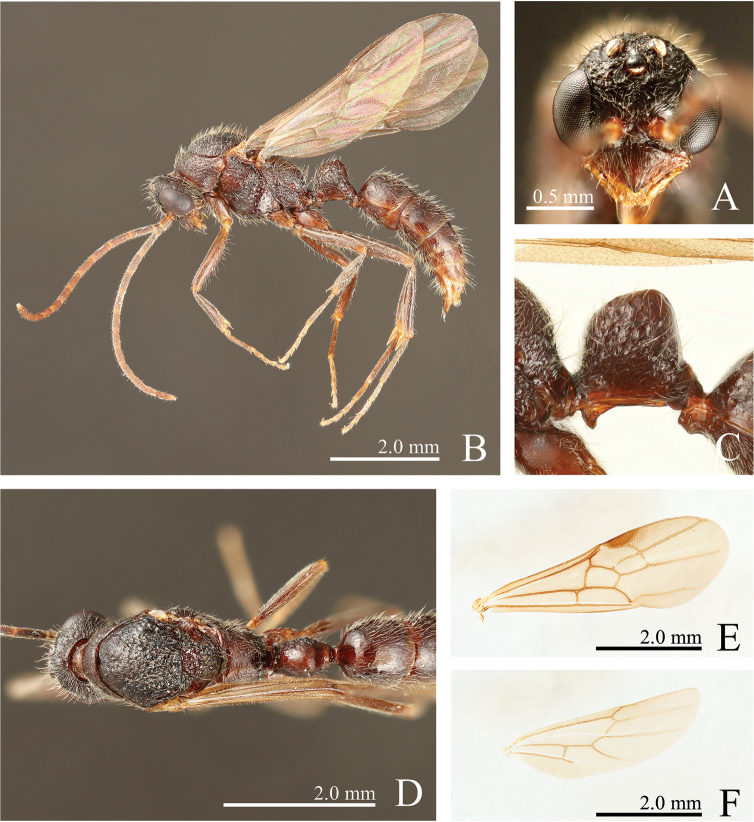
*Leptogenys itoi* sp. n., paratype, male. **A** head, full-face view **B** lateral view **C** petiole, lateral view **D** dorsal view **E** forewing **F** hindwing.

**Figure 11.**
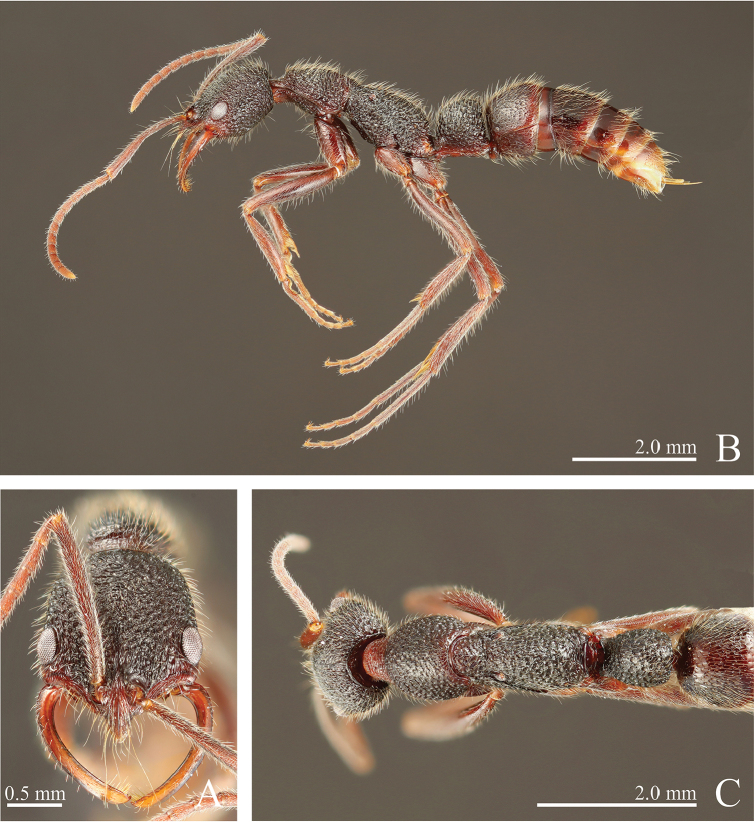
*Leptogenys kanaoi* sp. n., holotype, worker. **A** head, full-face view **B** lateral view **C** dorsal view.

**Figure 12.**
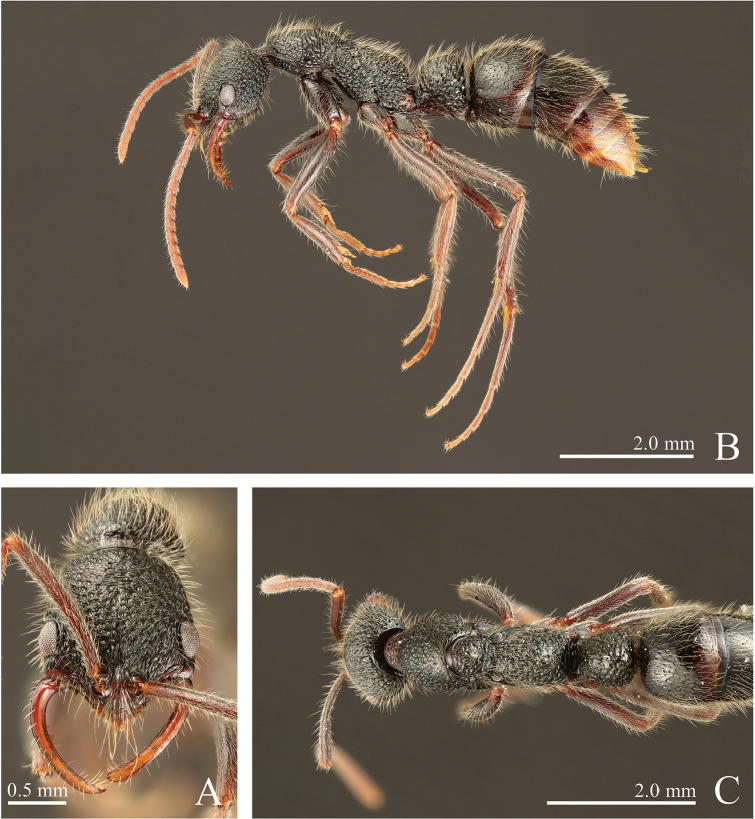
*Leptogenys kanaoi* sp. n., paratype, queen. **A** head, full-face view **B** lateral view **C** dorsal view.

**Figure 13.**
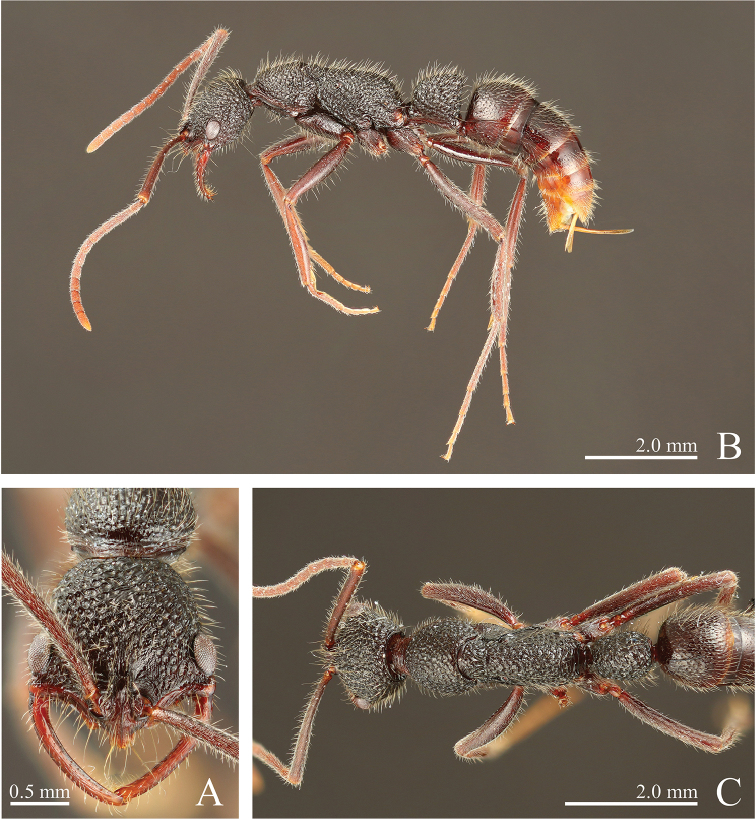
*Leptogenys malayana* sp. n., holotype, worker. **A** head, full-face view **B** lateral view **C** dorsal view.

**Figure 14.**
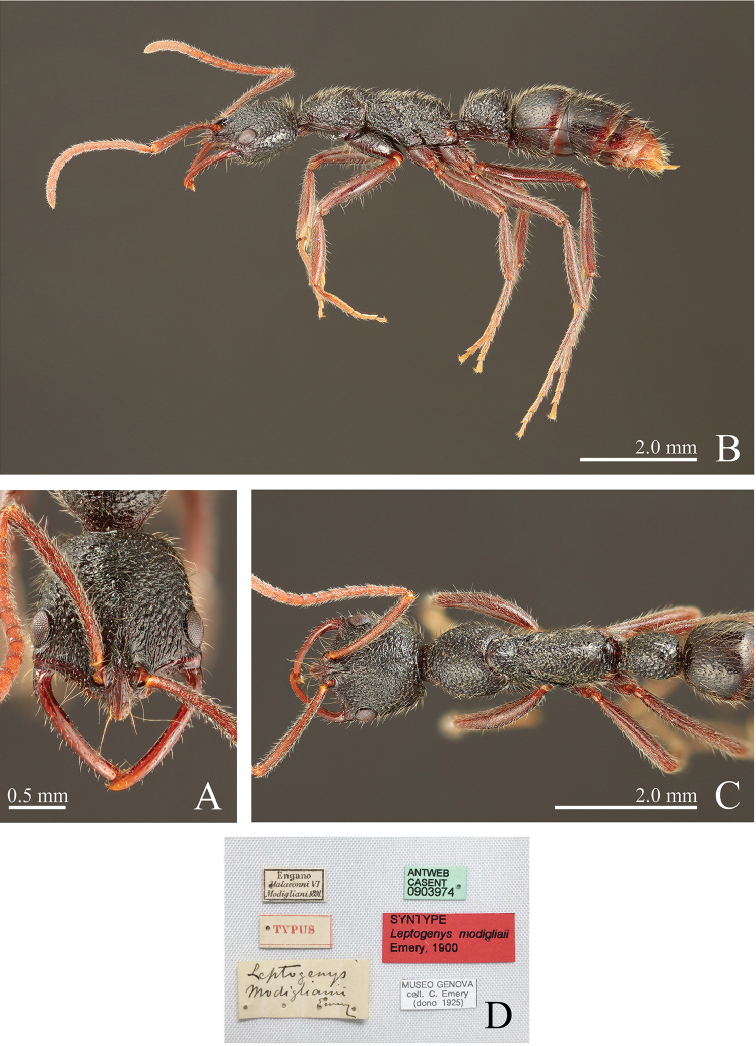
*Leptogenys modiglianii* Emery, 1900, lectotype, worker, specimen code: ANTWEB CASENT 0903974. **A** head, full-face view **B** lateral view **C** dorsal view **D** labels.

**Figure 15.**
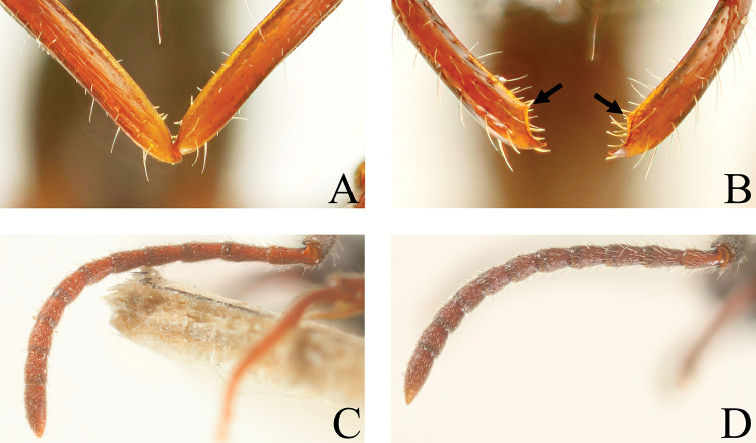
**A, B** Apex of mandible **C, D** Antenna. **A** *Leptogenys itoi*, subapical tooth absent **B** *Leptogenys breviloba*, subapical tooth present near apical tooth **C** *Leptogenys modiglianii*, antennoreme III ca. 2.3 times as long as wide **D** *Leptogenys breviloba*, antennoreme III ca. 1.6 times as long as wide.

**Figure 16.**
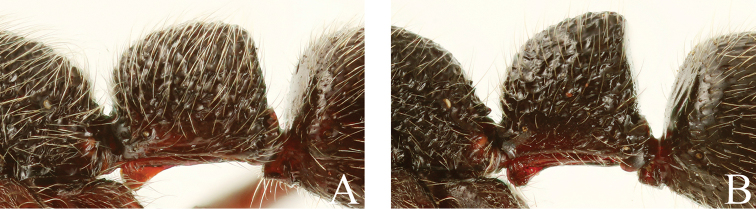
Petiole, lateral view. **A** *Leptogenys breviloba* **B** *Leptogenys itoi*.

**Figure 17.**
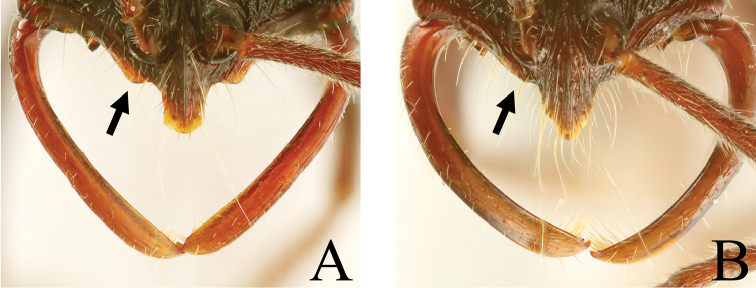
Clypeus and mandible, full-face view. **A** *Leptogenys itoi* **B** *Leptogenys kanaoi*.

**Figure 18.**
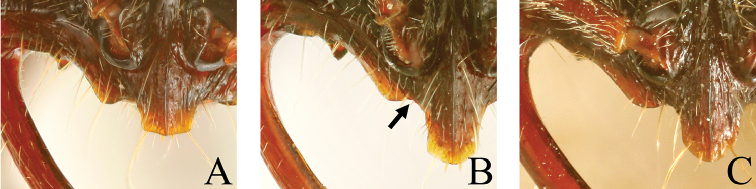
Clypeus, full-face view. **A** *Leptogenys malayana* **B** *Leptogenys itoi* **C** *Leptogenys modiglianii*.

**Figure 19.**
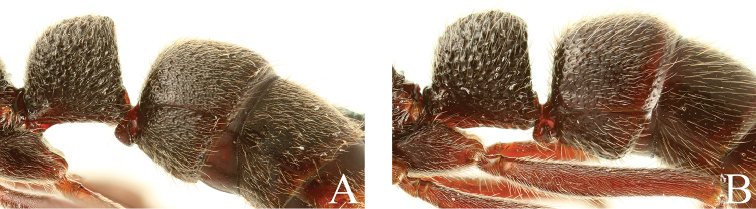
Petiole and gastar, lateral view. **A** *Leptogenys curva* **B** *Leptogenys kanaoi*.

### Key to species in the *Leptogenys modiglianii* species group from southeast Asia: worker

**Table:** 

1	In profile, petiole longer than or almost as long as high (LPI: 94–102); dorsal face of node subhorizontal (Fig. 16A)	***Leptogenys breviloba***
–	In profile, petiole higher than long (LPI: 103–122); dorsal face of node distinctly sloping anteriad (Fig. 16B)	**2**
2	In full-face view, head shorter (CI: 88–101). Mandible curved near base, becoming straight apically. Clypeus with lateral lobe (Fig. 17A: arrow)	**3**
–	In full-face view, head longer (CI: 77–83). Mandible distinctly curved throughout. Clypeus without lateral lobe (Fig. 17B: arrow)	**5**
3	In full-face view, head almost as long as wide (CI: 98–101). Antennal scape distinctly long (SL: 1.83–1.95; SI: 115–121), surpassing posterior margin of head by two-fifths of its length. Median clypeal extension with truncate apex (Fig. 18A)	***Leptogenys malayana***
–	In full-face view, head longer than wide (CI: 88–95). Antennal scape long (SL: 1.40–1.80; SI: 99–110), surpassing posterior margin of head by one-fifth to one-third of its length. Median clypeal extension with convex apex (Figs 18B, C)	**4**
4	In full-face view, head slightly longer than wide (CI: 93–95). Clypeus with blunt angle between lateral lobe and median extension (Fig. 18B: arrow). In profile, petiole distinctly higher than long (LPI: 109–118); in profile, anterior face of node ventrally vertical and dorsally forming continuous curve with dorsum	***Leptogenys itoi***
–	In full-face view, head distinctly longer than wide (CI: 88–90). Clypeus smoothly incurved between lateral lobe and median extension (Fig. 18C). In profile, petiole slightly higher than long (LPI: 103–108); in profile node with anterior and dorsal faces forming continuous curve	***Leptogenys modiglianii***
5	In profile, petiolar node with anterior and dorsal faces forming continuous curve. Gastral segment II covered with scalloped depressions, which close to each other. Body covered with understory layer of short dense hairs (Fig. 19A)	***Leptogenys curva***
–	In profile, anterior face of petiolar node ventrally vertical and dorsally forming continuous curve with dorsum. Gastral segment II smooth. Short hairs not forming understory layer. (Fig. 19B)	***Leptogenys kanaoi***

#### Leptogenys breviloba sp. n.

Taxon classificationAnimaliaHymenopteraFormicidae

http://zoobank.org/0697F839-348F-4C90-AEC7-C2CF2EBE68C3

Figures 3 , 4 , 5 , 15B , 15D , 16A

##### Etymology.

A combination of the Latin *brevis* (short), and the Latin *loba* (lobed), referring to the short clypeal lobe.

##### Type material.

**Holotype.** Worker (AK18), under the bark of rotted wood, secondary rainforest, Ulu Gombak, Gombak, Selangor, Malaysia, 3.32°N, 101.75°E, 230–350 m, 23 V 2012, Taku Shimada leg. [ELKU]. **Paratypes** (6 workers, 2 queens). 2 workers, same colony as the holotype [ELKU]; 4 workers, 2 queens (FI99-402), same place as the holotype, 20 VIII 1999, Fuminori Itô leg. [BLKU].

##### Type locality.

Ulu Gombak, Gombak, Selangor, Malaysia.

##### Distribution.

Malaysia: Peninsular Malaysia (Selangor).

##### *Worker*. Diagnosis.

In full-face view, head distinctly longer than wide (CI: 82–84). Mandible broadly curved throughout. Clypeus with lateral lobe, smoothly incurved between lateral lobe and median extension; apex of median extension convex. In profile, petiole longer than or almost as long as high (LPI: 94–102), highest near middle, with subhorizontal dorsal face.

##### Measurements

(n = 5, holotype in parentheses). HL: 1.35–1.42 (1.39), HLL: 1.07–1.09 (1.09), HLA: 0.12–0.16 (0.16), HW: 1.11–1.20 (1.14), CML: 0.18–0.21 (0.21), CI: 82–84 (82), CLI: 12–15 (15), SL: 1.32–1.38 (1.38), SI: 112–121 (121), EL: 0.34–0.37 (0.36), OI: 32–34 (33), PrL: 0.84–0.95 (0.84), PrH: 0.59–0.67 (0.67), PrW: 0.87–0.97 (0.90), WL: 2.22–2.37 (2.27), PeL: 0.85–0.88 (0.85), PeH: 0.81–0.89 (0.81), PeW: 0.60–0.67 (0.62), LPI: 94–102 (94), DPI: 71–76 (72).

##### Description.

In full-face view, head distinctly longer than wide. Mandible broadly curved throughout, with subapical tooth near apical tooth. Eye strongly prominent, measuring one-third of head lateral margin length. Antennal scape distinctly longer than width of head, surpassing posterior margin of head by one-fourth of its length; antennomere III ca. 1.6 times as long as wide. Clypeus with lateral lobe, smoothly incurved between lateral lobe and median extension; median extension short, with convex apex. In dorsal view, pronotum shorter than or slightly longer than wide. Metanotal groove weakly impressed. In profile, propodeal dorsum almost straight. Petiole in profile longer than or almost as long as high, highest near middle, with subrectangular node; anterior face of node ventrally vertical and dorsally forming continuous curve with dorsum; dorsal face subhorizontal, but weakly sloping anteriad; posterior margin almost straight to slightly concave.

Head distinctly areolate-rugose. Mandible smooth. Gastral segment I with scalloped depressions, which are irregular in size but generally small; distance between small depressions greater than diameter of depressions; large depressions close to each other; segments II smooth.

Body black, slightly tinged with red; clypeus, mandible, antenna, legs and ventral half of petiole dark-red. Apical two or three segments of gaster red-brown. Scalloped depressions on gastral segments I bearing hairs.

##### *Queen*. Measurements

(n = 2). HL: 1.32–1.46, HLL: 1.05–1.12, HLA: 0.15–0.16, HW: 1.14–1.21, CML: 0.21, CI: 83–87, CLI: 14–16, SL: 1.25–1.36, SI: 110–112, EL: 0.35–0.39, OI: 33–35, PrL: 0.88–0.98, PrH: 0.66–0.76, PrW: 0.94–0.98, WL: 2.27–2.41, PeL: 0.83–0.88, PeH: 0.87–0.95, PeW: 0.68–0.70, LPI: 105–108, DPI: 79–82. Mandible shorter than that of worker. Petiole in profile shorter than high, in dorsal view distinctly longer than wide.

##### *Male*.

Unknown.

##### Remarks.

The colony (AK18), including the holotype, was collected under the bark of rotted wood.

#### Leptogenys curva sp. n.

Taxon classificationAnimaliaHymenopteraFormicidae

http://zoobank.org/2FE7314C-1CBC-4D18-B13E-A2E6F1D9A491

Figures 3 , 6 , 7 , 19A

##### Etymology.

From the Latin *curvus*, meaning curved, referring to the curved mandible.

##### Type material.

**Holotype.** Worker (FI93-34), Sitiung, Dharmasraya, West Sumatra, Indonesia, I 1993, Fuminori Itô leg. [BLKU]. **Paratype.** 2 workers, 1 queen, same colony as the holotype [1 worker: BLKU; 1 worker, 1 queen: SKYC].

##### Type locality.

Sitiung, West Sumatra, Indonesia.

##### Distribution.

Indonesia (West Sumatra).

##### *Worker*. Diagnosis.

In full-face view, head distinctly longer than wide (CI: 77–78). Mandible distinctly curved throughout. Clypeus without lateral lobe; apex of median extension strongly projecting. In profile, petiole higher than long (LPI: 115–117), highest just anterior to posterodorsal angle; anterior and dorsal faces of node forming continuous curve; dorsal face distinctly sloping anteriad. Gastral segment II extensively covered with scalloped depressions. Body covered with understory layer of short, dense and recumbent pubescence.

##### Measurements

(n = 3, holotype in parentheses). HL: 1.71–1.75 (1.75), HLL: 1.18–1.22 (1.22), HLA: 0.14–0.15 (0.15), HW: 1.32–1.35 (1.35), CML: 0.29–0.31 (0.31), CI: 77–78 (77), CLI: 17–18 (18), SL: 1.43–1.52 (1.52), SI: 108–113 (113), EL: 0.34–0.36 (0.34), OI: 28–31 (28), PrL: 1.03–1.06 (1.03), PrH: 0.63–0.64 (0.64), PrW: 0.97–1.00 (0.97), WL: 2.48–2.53 (2.48), PeL: 0.81–0.82 (0.82), PeH: 0.93–0.95 (0.95), PeW: 0.65–0.67 (0.65), LPI: 115–117 (116), DPI: 80–82 (80).

##### Description.

In full face view, head distinctly longer than wide. Mandible distinctly curved throughout, with subapical tooth near apical tooth. Eye prominent, measuring one-fourth to one-third of head lateral margin length. Antennal scape longer than width of head, surpassing posterior margin of head by less than one-fifth of its length; antennomere III ca. 2.2 times as long as wide. Clypeus without lateral lobe; median extension moderately long, with apex strongly projecting. In dorsal view, pronotum longer than wide. Metanotal groove distinctly impressed. In profile, propodeal dorsum broadly convex. Petiole in profile higher than long, with fan-shaped node, highest just anterior to posterodorsal angle of node; anterior and dorsal faces of node forming continuous curve; dorsal face distinctly sloping anteriad; posterior face almost straight.

Head weakly areolate-rugose, longitudinally striate anteriorly; vertex weakly and transversally striate. Mandible longitudinally striate. Prosternum weakly rugose. Gastral segments I–II extensively covered with scalloped depressions that are irregular in size and close to each other; generally depressions on segment I larger than those on segment II.

Body black-gray; clypeus, mandible, antenna, legs, and ventral half of petiole dark-red. Body covered with understory layer of short, dense and recumbent pubescence. Scalloped depressions on gastral segments I–II bearing hairs.

##### *Queen*. Measurements

(n = 1). HL: 1.65, HLL: 1.19, HLA: 0.17, HW: 1.30, CML: 0.32, CI: 79, CLI: 20, SL: 1.41, SI: 109, EL: 0.36, OI: 31, PrL: 1.05, PrH: 0.75, PrW: 0.99, WL: 2.50, PeL: 0.76, PeH: 1.01, PeW: 0.74, LPI: 132, DPI: 97. Petiole in profile distinctly higher than long, in dorsal view almost as long as wide.

##### *Male*.

Unknown.

##### Remarks.

Gastral segments IV and V of the specimen examined were removed to confirm the genitalia by the collector.

#### Leptogenys itoi sp. n.

Taxon classificationAnimaliaHymenopteraFormicidae

http://zoobank.org/E07CBB4D-A7D1-4E99-98AF-F83BBB012EBF

Figures 3 , 8 , 9 , 10 , 15A , 16B , 17A , 18B

##### Etymology.

Dedicated to Professor Fuminori Itô, collector of the material examined.

##### Type material.

**Holotype.** Worker (FI99-240), transporting woodlouse, secondary rainforest, Ulu Gombak, Gombak, Selangor, Malaysia, 3.32°N, 101.75°E, 230–350 m, 3 VI 1999, Fuminori Itô leg. [BLKU]. **Paratypes** (47 workers, 6 queens, 2 male). 1 worker (FI92MG-270-5), same place as the holotype, VII-X 1992, Fuminori Itô leg. [BLKU]; 1 worker (FI92MG-270-7), same place, VII-X 1992, Fuminori Itô leg. [BLKU]; 2 workers, 1 queen (FI92MG-366), same place, VII-X 1992, Fuminori Itô leg. [1 worker, 1 queen: BLKU; 1 worker: SKYC]; 6 workers (FI92MG-456), 2 workers (FI92MG-463), same place, VII-X 1992, Fuminori Itô leg. [BLKU]; 4 workers (FI92MG-518), same place, VII-X 1992, Fuminori Itô leg. [3 worker: BLKU; 1 worker: SKYC]; 4 workers, 1 male (BG99-2), same place, V 1999, Bruno Gobin leg. [BLKU]; 4 workers (FI04-119), same place, 19 XII 2004, Fuminori Itô leg. [BLKU]; 6 workers, 1 queen, 1 male (FI04-120), same place, 17 XII 2004, Fuminori Itô leg. [BLKU]; 8 workers, 1 queen (AG05-20), same place, VII 2005, Ayako Gotô leg. [BLKU]; 1 worker, same place, 3 XII 2005, Seiki Yamane leg. [SKYC]; 3 workers, 1 queen (FI11-16), same place, 13 III 2011, Fuminori Itô leg. [BLKU]; 3 workers, 2 queens (FI11-17), same place, 13 III 2011, Fuminori Itô leg. [BLKU]; 1 worker, forest near road, Gap, Selangor, Malaysia, 900m, 14 III 1993, Löbl and Calame [MSNG]; 1worker, Fraser’s Hill, below Kuantun Ridge, Pahang, Malaysia, 1,350m, 17 III 1993, Löbl and Calame leg. [MSNG].

##### Type locality.

Ulu Gombak, Gombak, Selangor, Malaysia.

##### Distribution.

Malaysia: Peninsular Malaysia (Selangor and Pahang).

##### *Worker*. Diagnosis.

In full-face view, head slightly longer than wide (CI: 93–95). Mandible curved near base, becoming straight apically. Antennal scape long (SI: 99–110), surpassing posterior margin of head by two-fifths to one-third of its length. Clypeus with lateral lobe, with blunt angle between lateral lobe and median extension; median extension long, with broadly convex apex. In profile, petiole distinctly higher than long (LPI: 109–118), highest just anterior to posterodorsal angle; anterior face of node ventrally vertical and dorsally forming continuous curve with dorsum; dorsal face distinctly sloping anteriad.

##### Measurements

(n = 5, holotype in parentheses). HL: 1.66–1.73 (1.73), HLL: 1.12–1.18 (1.18), HLA: 0.13–0.15 (0.14), HW: 1.54–1.64 (1.64), CML: 0.29–0.36 (0.35), CI: 93–95 (95), CLI: 17–21 (20), SL: 1.57–1.80 (1.80), SI: 99–110 (110), EL: 0.31–0.35 (0.35), OI: 28–30 (30), PrL: 0.99–1.10 (1.10), PrH: 0.67–0.80 (0.79), PrW: 1.00–1.07 (1.05), WL: 2.47–2.72 (2.72), PeL: 0.84–0.95 (0.95), PeH: 0.99–1.04 (1.04), PeW: 0.68–0.74 (0.74), LPI: 109–118 (109), DPI: 76–84 (78).

##### Description.

In full face view, head slightly longer than wide. Mandible curved near base, becoming straight apically; subapical tooth obsolete. Eye prominent, measuring one-fourth to one-third of head lateral margin length. Antennal scape longer than width of head, surpassing posterior margin of head by two-fifths to one-third of its length; antennomere III ca. 2.5 times as long as wide. Clypeus with lateral lobe, with blunt angle between lateral lobe and median extension; median extension long, with broadly convex apex. In dorsal view, pronotum almost as long as wide. Metanotal groove slightly impressed. In profile, propodeal dorsum almost straight. Petiole in profile distinctly higher than long, highest just anterior to posterodorsal angle of node, with fan-shaped node; anterior face of node ventrally vertical and dorsally forming continuous curve with dorsum; dorsal face distinctly sloping anteriad; posterior face slightly concave dorsally and slightly convex ventrally.

Head strongly areolate-rugose, longitudinally striate near clypeus; vertex weakly and transversally striate. Mandible weakly striate longitudinally. Prosternum smooth and without punctures. Gastral segment I with scalloped depressions that are irregular in size; distance between depressions distinctly greater than diameter of depressions; interspace between depressions with small punctures; segments II smooth.

Body black, slightly tinged with red; clypeus, mandible, antenna, legs and ventral half of petiole dark-red. Apical two or three segments of gaster red-brown. Scalloped depressions on gastral segment I bearing hairs; small punctures without hairs.

##### *Queen*. Measurements

(n = 5). HL: 1.58–1.68, HLL: 1.07–1.14, HLA: 0.10–0.14, HW: 1.43–1.59, CML: 0.29–0.34, CI: 89–94, CLI: 18–20, SL: 1.51–1.62, SI: 102–107, EL: 0.30–0.36, OI: 27–31, PrL: 0.98–1.09, PrH: 0.66–0.79, PrW: 1.01–1.09, WL: 2.40–2.72, PeL: 0.84–0.90, PeH: 1.02–1.07, PeW: 0.72–0.78, LPI: 119–126, DPI: 85–90. Mandible shorter than that of worker. Petiole in profile distinctly higher than long, in dorsal view distinctly longer than wide.

##### *Male*. Measurements

(n = 2). HL: 1.08–1.17, HW: 0.83–0.87, CML: 0.35–0.38, CI: 75–77, CLI: 33, EL: 0.63–0.64, MeL: 1.01-1.10, MeW: 1.05–1.20, WL: 2.44–2.59, PeL: 0.68–0.75, PeH: 0.81–0.85, PeW: 0.54, LPI: 113–120, DPI: 71–80.

##### Description.

In full-face view, head longer than wide excluding eyes, wider posteriorly; posterior margin distinctly rounded. Mandible small, without basal angle and denticles, with rounded apex. Eye occupying over half of head length. Three prominent ocelli present; maximum length of lateral ocellus shorter than minimum distance between lateral ocellus and eye. Antenna 13-segmented; scape distinctly shorter than width of head; antennomere II shortest, roughly as long as wide; III longest; XIII shorter than III. Clypeus triangular and projecting anteriad, convex on midline; anterolateral margin straight; apex moderately acute; epistomal suture broadly rounded. Mesoscutum in profile convex, in dorsal view shorter than wide, widest posterior to mid-length; notaulus weakly notable; parapsidal line more than half of mesoscutal length; transscutal line almost straight. Anterior margin of mesoscutellar disc rounded. In profile, propodeal dorsum forming continuous curve with declivity; metapleural-propodeal sulcus present. Forewing ca. 3.0 times as long as wide, with stigma. Hindwing less than 0.8 times as long as forewing. Petiole in profile higher than long, in dorsal view longer than wide, highest posteriorly, with short peduncle, with fan-shaped node; anterior and dorsal faces forming convex line; dorsal face sloping anteriad; posterior face convex; in dorsal view, lateral margin broadly convex. With waist in profile, anterior lobe of subpetiolar process short-subtriangular, anterior margin convex, ventral apex moderately acute, posterior margin concave. With gaster in profile, prora low, long; continuously curved between prora and anteroventral edge of gastral sternite I. Constriction between gastral segments I and II distinct.

Head, mesosoma, and petiole weakly rugose, with piligerous small punctures. Mandible smooth. Clypeus with oblique to longitudinal striae. Subpetiolar process finely striate. Gastral segment I with irregularly sized depressions; distance between depressions distinctly greater than diameter of depressions; interspace between depressions with small punctures; segments II–V smooth; constriction between gastral segments I and II finely scrobiculate

Body black, tinged with red; clypeus, mandible, antenna, legs, subpetiolar process, and gaster red-brown. Hairs of various lengths, scattered, white-yellow; antennal scape and legs with dense short pubescence, mixed with long scattered hairs. Scalloped depressions and small punctures on gastral segment I bearing hairs.

##### Remarks.

This species corresponds to *Leptogenys* sp. 35 in Itô (1996). Workers selectively attack Isopoda, in which this species is thought to specialize (Itô 2000).

#### Leptogenys kanaoi sp. n.

Taxon classificationAnimaliaHymenopteraFormicidae

http://zoobank.org/6E7E651D-FCEF-445A-A775-F44FB961D35D

Figures 3 , 11 , 12 , 17B , 19B

##### Etymology.

Dedicated to Dr. Taisuke Kanao, collector of the material examined.

##### Type material.

**Holotype.** Worker (AK92), from rotted wood, mixed dipterocarp forest, Lambir Hills National Park, Miri, Sarawak, East Malaysia (Borneo), Malaysia, 4.20°N, 114.04°E, 90m, 4 X 2012, Taisuke Kanao leg. [FDS]. **Paratypes** (12 workers, 8 queens). 8 workers, 4 queens, same colony as the holotype [4 workers, 2 queens: FDS; 4 workers, 2 queens: ELKU]; 1 worker, Tower Region, same park, 16 VIII 1995, Seiki Yamane leg. [SKYC]; 3 workers, 4 queens (Eg98-BOR-868), Seipilok Forest, Sabah, East Malaysia (Borneo), Malaysia, 4 VII 1998, Katsuyuki Eguchi leg. [SKYC].

##### Type locality.

Lambir Hills National Park, Miri, Sarawak, East Malaysia (Borneo), Malaysia.

##### Distribution.

Malaysia: Borneo (Sarawak and Sabah).

##### *Worker*. Diagnosis.

In full-face view, head distinctly longer than wide (CI: 78–83). Mandible strongly curved throughout. Clypeus without lateral lobe; apex of median extension strongly projecting. Petiole in profile higher than long (LPI: 112–122), highest just anterior to posterodorsal angle; anterior face of node ventrally vertical and dorsally forming continuous curve with dorsum; dorsal face sloping anteriad. Gastral segment II smooth.

##### Measurements

(n = 5, holotype in parentheses). HL: 1.66–1.78 (1.77), HLL: 1.18–1.29 (1.24), HLA: 0.16–0.19 (0.19), HW: 1.34–1.43 (1.43), CML: 0.30–0.34 (0.34), CI: 78–83 (81), CLI: 17–19 (19), SL: 1.50–1.63 (1.56), SI: 109–116 (109), EL: 0.34–0.39 (0.36), OI: 27–30 (29), PrL: 1.04–1.09 (1.09), PrH: 0.69–0.74 (0.69), PrW: 1.03–1.07 (1.07), WL: 2.57–2.69 (2.69), PeL: 0.83–0.91 (0.91), PeH: 0.96–1.06 (1.02), PeW: 0.70–0.75 (0.75), LPI: 112–122 (112), DPI: 78–87 (82).

##### Description.

In full face view, head distinctly longer than wide. Mandible strongly curved throughout, with subapical tooth near apical tooth. Eye prominent, measuring one-fourth to one-third of head lateral margin length. Antennal scape distinctly longer than width of head, surpassing posterior margin of head by one-fifth of its length; antennomere III ca. 2.2 times as long as wide. Clypeus without lateral lobe; median extension moderately long, with apex strongly projecting. In dorsal view, pronotum longer than wide. Metanotal groove distinctly impressed. In profile, propodeal dorsum broadly convex. Petiole in profile higher than long, highest near mid-length, with roughly fan-shaped node; anterior face of node ventrally vertical and dorsally forming continuous curve with dorsum; dorsal face sloping anteriad; posterior face almost straight.

Head weakly areolate-rugose, longitudinally striate anteriorly; vertex weakly and transversally striate. Mandible weakly and longitudinally striate. Prosternum weakly rugose. Gastral segment I with scalloped depressions that are irregular in size and close to each other; segments II smooth.

Body black, gaster slightly tinged with red; clypeus, mandible, antenna, legs and ventral half of petiole dark-red. Apical two or three segments of gaster red-brown. Scalloped depressions on gastral segment I bearing hairs.

##### *Queen*. Measurements

(n = 5). HL: 1.59–1.74, HLL: 1.13–1.26, HLA: 0.16–0.18, HW: 1.30–1.39, CML: 0.26–0.33, CI: 80–82, CLI: 15–19, SL: 1.39–1.52, SI: 107–110, EL: 0.33–0.40, OI: 29–32, PrL: 0.96–1.03, PrH: 0.65–0.71, PrW: 1.00–1.06, WL: 2.39–2.56, PeL: 0.82–0.86, PeH: 1.03–1.09, PeW: 0.72–0.78, LPI: 123–126, DPI: 88–94. Petiole in profile distinctly higher than long, in dorsal view longer than wide.

##### *Male*.

Unknown.

##### Remarks.

The colony (AK92), including the holotype, was collected from rotted wood. It was composed of four queens, nine workers, and two pupae.

#### Leptogenys malayana sp. n.

Taxon classificationAnimaliaHymenopteraFormicidae

http://zoobank.org/AB930BCD-8634-46EB-86BE-7E9A74D75B30

Figures 3 , 13 , 18A

##### Etymology.

The species epithet, *malayana*, refers to the fact that this species was found on Peninsular Malaysia.

##### Type material.

**Holotype.** Worker, Trengganu, Malaysia, 1974, T. Clay leg. [BMNH]. **Paratypes.** 2 workers, same data as the holotype [BMNH].

Detailed collecting locality of the types is unknown. For convenience, the location of this species is marked at the center of Trengganu Province, Malaysia, on the distribution map (Fig. 3).

##### Type locality.

Trengganu, Malaysia.

##### Distribution.

Malaysia: Peninsular Malaysia (Trengganu).

##### *Worker*. Diagnosis.

In full-face view, head almost as long as wide (CI: 98–101). Mandible curved near base, becoming straight apically. Antennal scape distinctly longer than width of head (SI: 115–121), surpassing posterior margin of head by two-fifths of its length. Clypeus with lateral lobe, with blunt angle between lateral lobe and median extension; apex of median extension truncate. Petiole in profile distinctly higher than long (LPI: 112–116), highest just anterior to posterodorsal angle; anterior face of node ventrally vertical and dorsally forming continuous curve with dorsum; dorsal face distinctly inclined anteriad.

##### Measurements

(n = 3, holotype in parentheses). HL: 1.57–1.63 (1.63), HLL: 1.16–1.21 (1.18), HLA: 0.17–0.19 (0.17), HW: 1.59–1.61 (1.60), CML: 0.23–0.24 (0.23), CI: 98–101 (98), CLI: 14–15 (14), SL: 1.83–1.95 (1.91), SI: 115–121 (120), EL: 0.36–0.38 (0.37), OI: 31 (31), PrL: 1.01–1.09 (1.09), PrH: 0.74–0.89 (0.89), PrW: 1.12–1.14 (1.14), WL: 2.57–2.78 (2.78), PeL: 0.88–0.97 (0.97), PeH: 1.02–1.10 (1.09), PeW: 0.72–0.76 (0.76), LPI: 112–116 (112), DPI: 77–82 (78).

##### Description.

In full face view, head almost as long as wide. Mandible curved near base, becoming straight apically; subapical tooth distinct in holotype or absent in paratypes. Eye prominent, measuring one-third of head lateral margin length. Antennal scape distinctly longer than width of head, surpassing posterior margin of head by two-fifths of its length; antennomere III ca. 2.7 times as long as wide. Clypeus with lateral lobe, with blunt angle between lateral lobe and median extension; median extension short, with truncate apex. In dorsal view, pronotum shorter than wide. Metanotal groove slightly impressed. In profile, propodeal dorsum weakly convex to almost straight. Petiole in profile distinctly higher than long, highest just anterior to posterodorsal angle, with fan-shaped node; anterior face of node ventrally vertical and dorsally forming continuous curve with dorsum; dorsal face distinctly inclined anteriad; posterior face slightly concave dorsally and slightly convex ventrally.

Head strongly areolate-rugose, longitudinally striate near clypeus; vertex weakly and transversally striate. Mandible distinctly striate longitudinally. Gastral segment I with scalloped depression that are irregular in size but generally large; large depressions close to each other; distance between small depressions greater than diameter of depressions; segments II smooth.

Body black, gaster slightly tinged with red; clypeus, mandible, antenna, legs and ventral half of petiole dark-red. Apical two or three segments of gaster red-brown. Scalloped depressions on gastral segment I bearing hairs.

##### *Queen* and *male*.

Unknown.

#### Leptogenys modiglianii

Taxon classificationAnimaliaHymenopteraFormicidae

Emery, 1900

Figures 3 , 14 , 15C , 18C

Leptogenys modiglianii Emery, 1900: 13 (original description of workers; type locality: Malaconni, Engano Is., Indonesia); Bolton 1995: 232 (catalogue).
Leptogenys (Leptogenys) modiglianii Emery, 1900: Emery 1911: 100 (catalogue); Mann 1919: 298 (in key); Chapman and Capco 1951: 31 (checklist).

##### Type material.

**Lectotype** (present designation). Worker, Malaconni, Enggano Island, off the west coast of Sumatra, Indonesia, VI 1891, Elio Modigliani leg., specimen code: ANTWEB CASENT 0903974 [MSNG]. **Paralectotypes.** 4 workers, same data as the holotype [3 workers: MSNG; 1 worker: NHMW].

##### Distribution.

Indonesia: Enggano Island.

##### *Worker*. Diagnosis.

In full-face view, head distinctly longer than wide (CI: 88–90). Mandible curved near base, becoming straight apically. Antennal scape slightly longer than width of head (SI: 103–105), surpassing posterior margin of head by one-fifth to one-fourth of its length. Clypeus with lateral lobe, smoothly incurved between lateral lobe and median extension; median extension with convex apex. In profile, petiole slightly higher than long (LPI: 103–108), highest just anterior to posterodorsal angle; anterior and dorsal faces forming continuous curve; dorsal face distinctly sloping anteriad.

##### Measurements

(n = 5, lectotype in parentheses). HL: 1.52–1.68 (1.68), HLL: 1.11–1.22 (1.22), HLA: 0.18–0.20 (0.18), HW: 1.34–1.50 (1.50), CML: 0.25–0.28 (0.28), CI: 88–90 (90), CLI: 15–17 (17), SL: 1.40–1.58 (1.58), SI: 103–105 (105), EL: 0.30–0.34 (0.34), OI: 26–28 (28), PrL: 0.99–1.09 (1.09), PrH: 0.59–0.75 (0.75), PrW: 0.95–1.02 (1.02), WL: 2.49–2.68 (2.68), PeL: 0.85–0.92 (0.92), PeH: 0.91–0.98 (0.98), PeW: 0.64–0.67 (0.67), LPI: 103–108 (106), DPI: 73–76 (73).

##### Redescription.

In full-face view, head distinctly longer than wide. Mandible curved near base, becoming straight apically; subapical tooth absent. Eye prominent, measuring one-fourth to one-third of head lateral margin length. Antennal scape slightly longer than width of head, surpassing posterior margin of head by one-fifth to one-fourth of its length; antennomere III ca. 2.3 times as long as wide. Clypeus with lateral lobe, smoothly incurved between lateral lobe and median extension; median extension moderately long, with convex apex. In dorsal view, pronotum longer than wide. Metanotal groove weakly impressed. In profile, propodeal dorsum almost straight. Petiole in profile slightly higher than long, highest just anterior to posterodorsal angle, with fan-shaped node; anterior and dorsal faces forming continuous curve; dorsal face distinctly sloping anteriad; posterior face almost straight but slightly convex.

Head weakly areolate-rugose, longitudinally striate anteriorly; vertex weakly and transversally striate. Mandible smooth. Gastral segment I with scalloped depressions that are irregular in size; distance between depressions distinctly greater than diameter of depressions; interspace between depressions with small punctures; segment II smooth.

Body black, slightly tinged with red; clypeus, mandible, antenna, legs and lower half of petiole dark-red. Apical two or three segments of gaster red-brown. Scalloped depressions on gastral segment I bearing hairs, small punctures without hairs.

##### *Queen* and *male*.

Unknown.

## Discussion

In the Oriental region, the members of the *Leptogenys modiglianii* species group are similar to those of the *Leptogenys maxillosa* species group, which includes two species, *Leptogenys falcigera* Roger, 1861 and *Leptogenys pruinosa* Forel, 1900 in the region. They share several features such as the elongate and curved mandible, anterior edge of torulus located anterior to mandible base, short clypeus, eye situated just posterior to clypeus and breaking outline of lateral margin of head, hypostomal teeth visible in full-face view, petioler node subrectangular in profile, and subpetiolar process consisting of high anterior lobe and thin posterior extension. However, the *Leptogenys modiglianii* species group is distinguished from the *Leptogenys maxillosa* group by the following contrasting character states (*maxillosa* group in parentheses): clypeus with apical median extension (without median extension), body surface mostly areolate-rugose (with shagreened sculpture), and body extensively covered with standing hairs (without standing hairs).

Mann (1919) indicated that *Leptogenys modiglianii* is related to four species in the Australian region: *Leptogenys emeryi* Forel, 1901 from Bismarck Islands, *Leptogenys foreli* Mann, 1919 from Solomon Islands, *Leptogenys triloba* Emery, 1901 from New Guinea, and *Leptogenys truncate* Mann, 1919 from Santa Cruz Islands. These four species share the above diagnostic characters with the *modiglianii* species group defined in this paper and possibly belong to this species group.

There is little information about this species group. Most of the specimens examined in this study were collected in one place: Ulu Gombak on the Peninsular Malaysia. This species group may also occur in Java, Kalimantan, and Sulawesi, Indonesia, and additional as-yet undescribed species may be found in Sundaland and New Guinea.

This species group in southeast Asia is divided into three subgroups. One, which contains *Leptogenys itoi*, *Leptogenys malayana* and *Leptogenys modiglianii*, is recognized by elongate antennomere III that is ca. 2.3–2.7 times as long as wide (Fig. 15C), presence of the lateral lobe on the anterior margin of the clypeus (Fig. 17A), short petiole (LPI: 103–118), and fan-shaped petiolar node. The second group, which contains *Leptogenys kanaoi* and *Leptogenys curva*, is recognized by the strongly curved mandible, relatively short antennomere III that is ca. 2.2 times as long as wide, absence of the lateral lobe on the anterior margin of the clypeus (Fig. 17B), short petiole (LPI: 112–122), and fan-shaped petiolar node. *Leptogenys breviloba* is a remarkable species among this species group, with very short antennomere III that is ca. 1.6 times as long as wide (Fig. 15D), anterior margin of the clypeus with lateral teeth, long petiole (LPI: 94–102), and rectangular petiolar node.

## Supplementary Material

XML Treatment for Leptogenys breviloba

XML Treatment for Leptogenys curva

XML Treatment for Leptogenys itoi

XML Treatment for Leptogenys kanaoi

XML Treatment for Leptogenys malayana

XML Treatment for Leptogenys modiglianii
